# Comparison of Features for Movement Prediction from Single-Trial Movement-Related Cortical Potentials in Healthy Subjects and Stroke Patients

**DOI:** 10.1155/2015/858015

**Published:** 2015-06-16

**Authors:** Ernest Nlandu Kamavuako, Mads Jochumsen, Imran Khan Niazi, Kim Dremstrup

**Affiliations:** ^1^Department of Health Science and Technology, Aalborg University, 9220 Aalborg, Denmark; ^2^Center for Chiropractic Research, New Zealand College of Chiropractic, Auckland 1060, New Zealand; ^3^Faculty of Health & Environmental Sciences, Health & Rehabilitation Research Institute, AUT University, Auckland 1060, New Zealand

## Abstract

Detection of movement intention from the movement-related cortical potential (MRCP) derived from the electroencephalogram (EEG) signals has shown to be important in combination with assistive devices for effective neurofeedback in rehabilitation. In this study, we compare time and frequency domain features to detect movement intention from EEG signals prior to movement execution. Data were recoded from 24 able-bodied subjects, 12 performing real movements, and 12 performing imaginary movements. Furthermore, six stroke patients with lower limb paresis were included. Temporal and spectral features were investigated in combination with linear discriminant analysis and compared with template matching. The results showed that spectral features were best suited for differentiating between movement intention and noise across different tasks. The ensemble average across tasks when using spectral features was (error = 3.4 ± 0.8%, sensitivity = 97.2 ± 0.9%, and specificity = 97 ± 1%) significantly better (*P* < 0.01) than temporal features (error = 15 ± 1.4%, sensitivity: 85 ± 1.3%, and specificity: 84 ± 2%). The proposed approach also (error = 3.4 ± 0.8%) outperformed template matching (error = 26.9 ± 2.3%) significantly (*P* > 0.001). Results imply that frequency information is important for detecting movement intention, which is promising for the application of this approach to provide patient-driven real-time neurofeedback.

## 1. Introduction

The movement-related cortical potential (MRCP) is a slow negative brain potential that can be observed in the electroencephalogram (EEG) up to 2 s prior to self-initiated movements: self-paced and cue-based [1, 2]. In addition, MRCPs can be observed in the EEG with a similar wave form when self-initiated movements are imagined [3]. Due to the intrinsic feature that a depression can be observed in the EEG prior to an imaginary movement, the MRCP has been used as a control signal in brain-computer interface (BCI) technology. BCIs can link the activity in the brain with an external device through a series of steps that include preprocessing, for example, the electrical activity to enhance the signal-to-noise ratio, extracting features to discriminate between two or more different states which then can be translated to device commands by a classifier [4]. BCIs have mainly been used for control and communication purposes [4], but in recent years, its potential in neurorehabilitation has been explored [5]. The MRCP has been suggested in several studies to be a useful control signal for BCIs in neurorehabilitation [4, 5]. The movements or attempted movements from motor impaired patients can be predicted around 100 ms before their onset, which is important for induction of Hebbian-like plasticity (an underlying mechanism for motor recovery) [6], which leaves time to close the disrupted motor control loop by initiating electrical stimulation or rehabilitation robots [7–11]. Besides the MRCP, different approaches to detect movement intentions for BCI for neurorehabilitation exist. These include, for example, extraction of spectral components from, for example, power spectral densities and sensorimotor rhythms [12–15].

In a previous study it was shown that the outcome of a BCI-based rehabilitation protocol was positively correlated with the performance of the system [9]; therefore, there is an incitement for optimizing BCI system performance. As outlined, the BCI consists of different components (preprocessing, feature extraction, and classification) that may be optimized using various signal processing techniques. Previously proposed preprocessing methods include temporal, spectral, and spatial filtering as well as blind source separation [8, 16–19]. For decoding of movement intention using MRCPs and other control signals, two strategies have been used in the literature, detection and classification. For example, for detection, a matched filtering [8] approach can be used to detect the initial negative phase of MRCP with help of template matching. Using classification methods, it has to be set up as a 2-class classification problem between two states: an idle state (often noise) and an active state (motor execution or motor imagination).

From the raw EEG signal point of view, various features and classifiers have been applied in BCI for control purposes [15, 16] using different classifiers [9, 11, 13, 14, 17]. In rehabilitation, the focus has been on spectral features computed from the EEG around the intended movement onset [15] or from temporal features computed from the MRCP (low pass filtering) usually detected by a simple matched filter (MF) [7] from a single optimized channel. The application of MF is optimal when the noise is additive Gaussian, a condition that usually fails in real applications [9]. Recently, application of locality preserving projections (LPP) has proven suitable for the detection of MRCPs [9]. Although LPP proved superior compared to MF, the lower dimensional feature space does not provide information about the characteristics of the signals that maximize separability between movement and noise. Furthermore, 0.5 s after the movement onset was also included during training of the detector, and as for any dimension reduction algorithms, the optimal dimension for the LPP space needs to be determined to achieve optimal performance. In this study, we compare the detection performance of MF with time domain features extracted from the initial negative phase of the MRCP and spectral features computed from the entire band of EEG, which have shown to be useful for movement discrimination, for example, using sensorimotor rhythms. Thus we propose to compare matched filtering with features extracted from the frequency domain of EEG (full band) and from the time domain of the MRCP recorded in cue-based sessions to discriminate between two states: no movement (idle state) and movement intention (using only information prior to the movement onset) with the constraint that movements must be predicted to fulfill the temporal association of induction of Hebbian-like plasticity. The investigation was conducted on real, imaginary, and attempted (stroke patients) movements with linear discriminant analysis (LDA) for classification.

## 2. Methods

### 2.1. Subjects

24 healthy subjects (7 women and 17 men: 27 ± 4 years old) and six stroke patients with lower limb paresis participated in the study. All procedures were approved by the local ethical committee (N-20100067 and N-20130081), and the participants gave their written informed consent before the experiment.

### 2.2. Experimental Protocol

**Figure 1:**
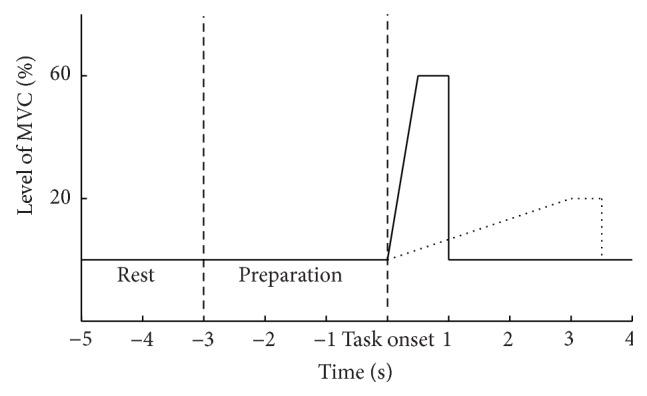
The subjects were presented with the following cues for each of the two tasks. In the 3 s prior to the task onset subjects were asked to start preparing to perform the motor execution or imagination. They rested after the movement was maintained for 0.5 s.

**Figure 2:**
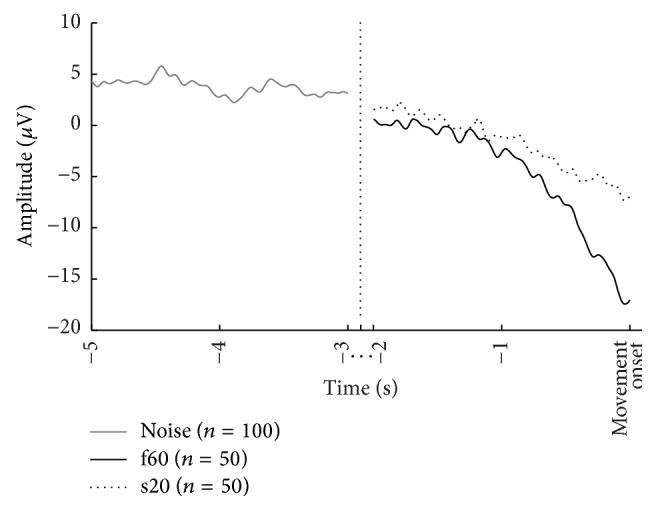
Ensemble average of the epochs associated with the two movement tasks and the noise for a healthy subject performing the actual movement.

The subjects sat comfortably in a chair. After placement of the EEG electrodes, the right leg (or affected for the patients) was fixed to a custom-made pedal for ankle joint torque measurements. Subjects performed maximum voluntary contraction (MVC) three times and the highest value was retained to compute 20% and 60% MVC. After the MVC was determined, subjects performed 2 × 50 repetitions (see Figure 1) of the following tasks of ankle dorsiflexion: (1) a fast movement (f60: 0.5 s to reach 60% MVC) and (2) a slow movement (s20: 3 s to reach 20% MVC) [20]. The ensemble average of the movements is shown in Figure 2.

Subjects were provided with a visual cue (the two traces in Figure 1) in order to perform the movements correctly as they were constrained to spend the given time to reach the desired level of MVC. The healthy subjects were randomly divided into two subgroups of 12. One group was asked to perform the actual dorsi-flexion movements while the subjects in the other group imagined the movements. The patients were asked to attempt to perform the tasks. The tasks were randomized in blocks, and ~5 min practice was performed before each task.

### 2.3. Signal Acquisition

#### 2.3.1. EEG

Continuous EEG was recorded from FP1, F3, Fz, F4, C3, Cz, C4, P3, Pz, and P4 according to the international 10–20 system (32 Channel Quick-Cap, Neuroscan and EEG Amplifiers, Numaps Express, Neuroscan). The signals were referenced to the right ear lobe. The EEG was sampled with 500 Hz and digitized with 32 bits accuracy. Electrooculography (EOG) was registered from FP1. The impedance of the electrodes was below 5 kΩ during the experiment. The digital trigger from the interface software was sent to the EEG amplifier to be used as marker to segment the continuous EEG into epochs.

#### 2.3.2. Force Measurement

For real movements, force was used as input to the program that cued the subjects, so they were provided with visual feedback on their performance. Subjects performing imaginary movement did also receive a cue, but the force signal was only used to verify that the movement was not actually performed. The force was recorded with custom-made software (SMI, Aalborg University) and sampled with 2000 Hz. The MVC was recorded at the beginning of the experiment. The onset of each executed movement for the healthy subjects and patients was determined from the force trace. This was used to synchronize all epochs. It was identified as the first sample when all values in a 200 ms window (with a 1-sample shift) exceeded the baseline. The baseline was defined as the mean value of the signal 2–4 s before the task onset provided by the visual cue.

### 2.4. Signal Processing

The EEG was band-pass filtered with a 2nd order Butterworth filter from 0.05–10 Hz in the forward and reverse direction for the extraction of temporal features. The spectral features were extracted from data that was high-pass filtered with a cut-off frequency of 0.05 Hz. To correct for the poor spatial resolution of EEG, the data was spatially filtered using a large Laplacian spatial filter, so a single surrogate channel was obtained (linear combination of the nine EEG channels).

Signal epochs were extracted from the movement/task onset and 2 s prior to this point; noise epochs were extracted 5–3 s prior to the movement/task onset from the surrogate channel.

The proposed approach of movement detection consisted of two steps: a feature extraction step from each epoch and a classification (signal versus noise epoch) step using LDA. This approach was compared to the method based on template matching as previously proposed [8, 9, 20, 21]. In short, the template matching technique was based on determining a suitable MRCP template and used correlation measures to detect a similar shape in the testing data. All the features and the template matching were performed on the surrogate channel.

### 2.5. Feature Extraction

#### 2.5.1. Temporal Features

Six temporal features were extracted for the executed movements, as described previously [20]: (i) point of maximum negativity, (ii) mean amplitude, (iii + iv) slope and intersection of a linear regression of the data until the point of detection, and (v + vi) slope and intersection of a linear regression of the data from the movement onset and 0.5 s prior to this point. For the imaginary movements, the same features were extracted except for the intersections of the two linear regressions. Also, the mean amplitude of the data from the movement onset and 0.5 s prior to this point was extracted.

#### 2.5.2. Spectral Features

Five spectral features were extracted. Welch's power spectral density estimate was calculated on each epoch using a Hamming window with 50% overlap of the segments. The average power was calculated in the following frequency ranges: (i) 0–4 Hz, (ii) 4–8 Hz, (iii) 8–13 Hz, (iv) 13–30 Hz, and (v) 30–100 Hz. These ranges correspond approximately to the delta, mu, alpha, beta, and gamma frequency bands, respectively.

### 2.6. Movement Detection

We introduce the concept of 5-fold test procedure to evaluate the proposed classifier-based approach. N-fold test differs from N-fold validation as N-fold test includes (N-1)-fold validation step. The procedure is as follows:

1. Divide data into five parts (part1, part2, part3, part4, and part5).
2. Use four parts (e.g., part1, part2, part3, and part4) in a 4-fold validation procedure where three parts are used for training and the remaining part is used for validation in order to minimize overfitting. For the classifier approach, this step finds the best LDA structure. For the template matching approach, the best template, with associated best threshold, is determined.
3. Permute and repeat item 2 four times.
4. Find the training set with the lowest validation error (e.g., part2).
5. Use this best training set to test on the last part (not used in the validation step, here part5).
6. Permute and repeat from item 2 five times.
7. Report the average test error.

This procedure was performed for each feature type (temporal and spectral) to compare their ability to discriminate between noise and movement.

### 2.7. Performance Measures

The performance of the system was quantified using the detection error, sensitivity, and specificity. Detection error is the sum of false positives and false negatives. Sensitivity (also called true positive rate) measures the proportion of actual positives (movements) which are correctly identified. Specificity (sometimes called the true negative rate) measures the proportion of negatives (no motion or noise) which are correctly identified.

### 2.8. Statistical Analysis

The statistical analysis was carried out for each task separately first by comparing temporal and spectral features and then comparing the best of these two to template matching. Thus for each task (real, imaginary, and attempted movement), the nonparametric Friedman's test was used to evaluate the difference between spectral and temporal features and the difference between the best feature type and template matching approach. *P* values less than 0.05 were considered significant. Results are presented as mean ± standard error.

## 3. Results

**Figure 3:**
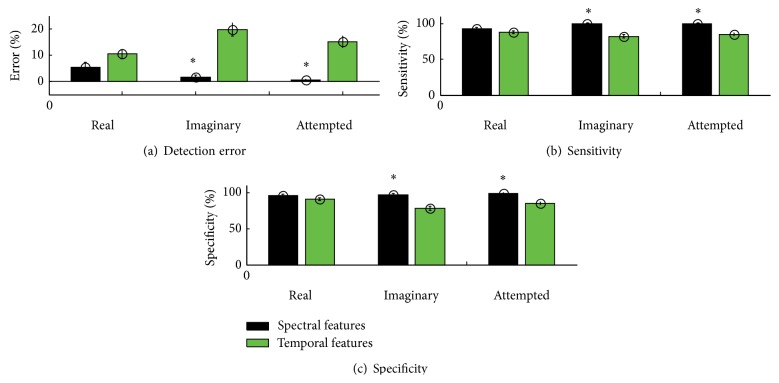
Detection performance of different features in terms of (a) detection error, (b) sensitivity, and (c) specificity for the three tasks. *∗* indicates where the difference is statistically significant.

The ensemble average across tasks when using spectral features was (error: 3.4 ± 0.8%, sensitivity: 97.2 ± 0.9%, and specificity: 97 ± 1%) significantly better (*P* < 0.01) than temporal features (error: 15 ± 1.4%, sensitivity: 85 ± 1.3%, and specificity: 84 ± 2%). However, for each single task, the difference was significant only when using imaginary and attempted movements (*P* < 0.005) suggesting that temporal features are discriminative only when the movement is actually performed.  Figure 3 summarizes the results.

**Figure 4:**
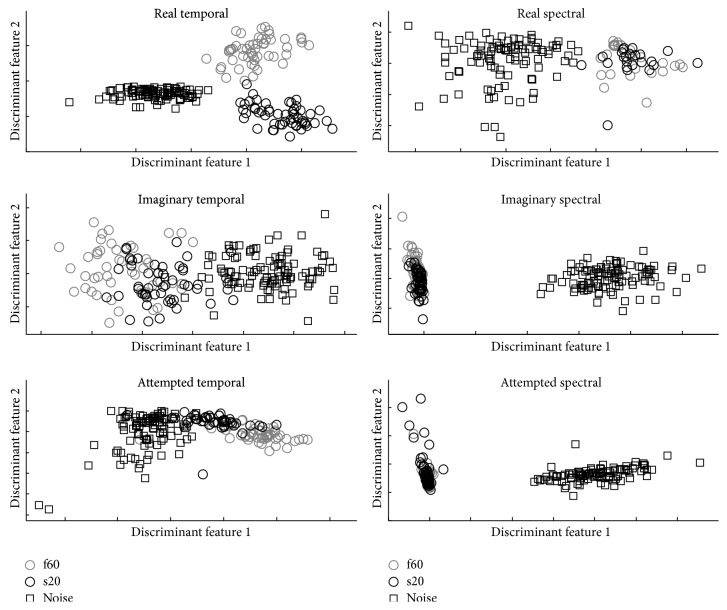
Feature space representation using two discriminative features after Fisher projection. Features are normalized between −1 and 1 for the sake of clarity for the best subject at each task. Squares represent noise, black circles represent s20, and gray circles represent f60.

**Figure 5:**
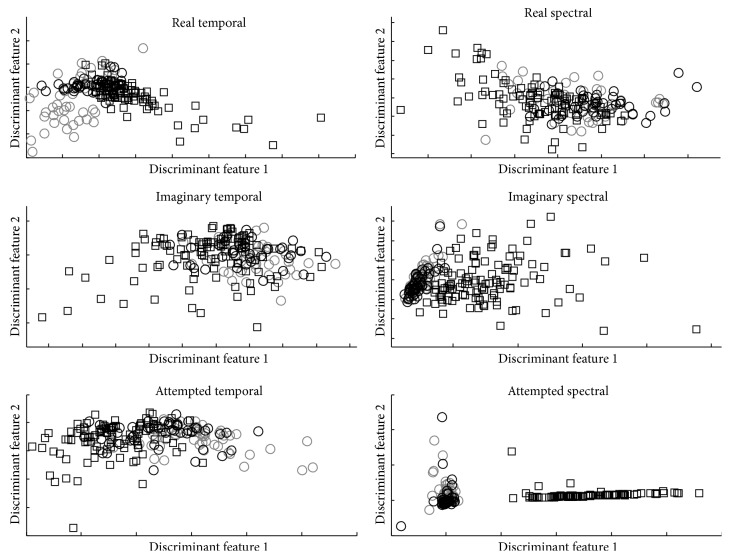
Feature space representation using 2 discriminative features after Fisher projection. Features are normalized between −1 and 1 for the sake of clarity for the worst subject at each task. Square represents noise, black circles represent s20, and gray circles represent f60.

Figures 4 and 5 show the two discriminative features when each feature type is projected using Fisher discrimination projection considering three classes (f60, s20, and noise) for the best subject and worst subject, respectively.

For all tasks the proposed classifier-based approach outperformed the template matching approach significantly (*P* < 0.001) suggesting that the use of mainly temporal information of the MRCP is not optimal for detection purposes. The results for template matching and spectral features are summarized in Table 1.

## 4. Discussion

Spectral features of EEG and MRCP temporal features were investigated to test the performance of a BCI that can detect movement intention and this was also compared with MF. Spectral features classified by LDA led to a lower detection error compared to temporal features and a template matching technique using the noise and signal epochs containing MRCP.

### 4.1. LDA Classification of Temporal and Spectral Features for Movement Detection and Template Matching

Overall classification accuracies that were obtained for the spectral features were higher compared to those obtained when using the temporal features across the tasks (real, imaginary, and attempted). This finding suggests that the spectral features are less sensitive to the variability in the signal compared to, for example, the amplitude of specific segments of the MRCP. However on further exploration it was found that the difference between temporal and spectral features classification was not significant for real movements (Figure 3) as compared to imaginary or attempted movement of stroke patients. This can be explained by the greater signal-to-noise ratio that is observed for motor execution [3, 20]. The risk that the MRCPs are corrupted by muscle or movement artifacts is limited due to the higher frequency content compared to the MRCP frequency range. The usefulness of a BCI operated by motor execution can be debated since EMG is better for, for example, prosthetic control due to higher signal-to-noise ratio. However, in applications where early detection of movement intentions is essential, such as neurorehabilitation, EEG can be used to predict when a movement occurs before the onset of EMG (movement). The performance of the classifier to estimate the detection performance was higher compared to previous studies where the detection performance was estimated from noise-signal discrimination [18, 19, 22–27]. It should be noted, however, that similar movements were not performed in the different studies leading to differences in signal morphology and signal-to-noise ratios. The optimal feature type (spectral) was compared to template matching which is one of the current techniques that have been implemented in online BCI for neurorehabilitation purposes [9]. It was found that the use of spectral features outperformed the template matching technique. The performance of the template matching technique is similar as reported in previous studies [8–10, 21, 28] using simulated or actual online systems. Contrary to projection-based feature selection [9], the current study reveals the actual features that are needed for improved performance, thus eliminating the need for parameter optimization for each use.

In the current study a single Laplacian channel was used based on a limited number of electrodes (nine). It should be investigated if performing the same feature extraction on all nine electrodes could lead to better detection performance. This will lead to a feature vector of higher dimensionality where only some of the features will be useful. Therefore, different feature selection methods should be investigated such as principal component analysis or sequential forward feature selection techniques. This could potentially improve the detection performance.

### 4.2. Implications

There is no consensus to what type of performance is good for rehabilitation systems [23] based on BCI. There is some evidence that performance might be related to outcome measure of neurorehabilitation [8, 24]. For this reason, epochs extracted from cue-based MRCPs were used to reduce the variability in performance results as compared to the results obtained in self-paced MRCPs. Improving the decoding of movement-related activity from the brain means that a causal link can be established between the brain and muscles in the weakened part of the body when combined with assistive technologies for, for example, stroke rehabilitation. Besides restorative interventions, improved control can be useful for controlling, for example, prosthetic devices in patients that are severely damaged, where myoelectric control is not possible; this, however, will most likely require more degrees of freedom than a binary switch.

### 4.3. Limitations

The analysis in this study was performed offline on the contrary to the intended use, which is online. However, the proposed techniques for feature extraction and classification are simple to use and are expected to be easily implemented in an online system. The use of the zero-phase shift filter has been implemented previously [29]. To implement the filter in an online system the data must be imported in blocks and streamed continuously; however, this processing delay is expected to be only a few milliseconds, since it is a 2nd order filter. Alternatively, a Butterworth filter could be used without the zero-phase shift implementation, since the phase shift in the passband is linear and the processing delay is expected to be low. The performance of the classifier is expected to decrease due to the fact that movement epochs in this study were extracted with a priori knowledge of when the movement occurred; therefore, the optimal 2 s data window could be extracted which will not be the case in an online system where incoming data will be classified continuously. Another aspect that must be taken into consideration is how often the incoming data should be processed in an online system; if the delay is small between two consecutive classifications, the system will be activated many times around each movement where the features are likely to be similar. However, this may be avoided by using a majority vote of X consecutive windows or by increasing the delay between the data segments that are classified.

## 5. Conclusions

This study compared the use of spectral features with time domain features derived from the EEG and template matching for improved detection of movement intentions in offline study. The spectral features outperformed significantly the temporal features for imaginary and attempted movements but for real movements it was not significant. Furthermore, spectral feature-based classification also outperformed significantly the template matching approach. The results imply that frequency information is important for detecting movement intentions, which is promising for the application of this approach to provide patient-driven real-time neurofeedback.

## Figures and Tables

**Table 1:** Result summary when comparing the proposed classifier approach against template matching. Results are given as mean ± SE and are all expressed in %.

	Spectral features	Template matching
Real movement		
Detection error	5.5 ± 1.8	28.9 ± 3.5
Sensitivity	93.0 ± 1.7	82.0 ± 2.7
Specificity	96.0 ± 2.1	60.0 ± 5.0
Imaginary movements		
Detection error	1.5 ± 0.83	29.6 ± 2.8
Sensitivity	100 ± 0	80.6 ± 2.5
Specificity	97 ± 1.7	60.3 ± 4.2
Attempted movements		
Detection error	0.63 ± 0.21	22.4 ± 2.4
Sensitivity	99.8 ± 0.17	83.8 ± 1.7
Specificity	98.9 ± 0.39	71.3 ± 4.6
